# PSGL-1 Is a T Cell Intrinsic Inhibitor That Regulates Effector and Memory Differentiation and Responses During Viral Infection

**DOI:** 10.3389/fimmu.2021.677824

**Published:** 2021-07-13

**Authors:** Roberto Tinoco, Emily N. Neubert, Christopher J. Stairiker, Monique L. Henriquez, Linda M. Bradley

**Affiliations:** ^1^ Department of Molecular Biology and Biochemistry, University of California, Irvine, Irvine, CA, United States; ^2^ Infectious and Inflammatory Disease Center, NCI Designated Cancer Center, Sanford Burnham Prebys Medical Discovery Institute, La Jolla, CA, United States

**Keywords:** virus infection, PSGL-1, effector T cells, memory T cells, LCMV

## Abstract

Effective T cell differentiation during acute virus infections leads to the generation of effector T cells that mediate viral clearance, as well as memory T cells that confer protection against subsequent reinfection. While inhibitory immune checkpoints have been shown to promote T cell dysfunction during chronic virus infections and in tumors, their roles in fine tuning the differentiation and responses of effector and memory T cells are only just beginning to be appreciated. We previously identified PSGL-1 as a fundamental regulator of T cell exhaustion that sustains expression of several inhibitory receptors, including PD-1. We now show that PSGL-1 can restrict the magnitude of effector T cell responses and memory T cell development to acute LCMV virus infection by limiting survival, sustaining PD-1 expression, and reducing effector responses. After infection, PSGL-1-deficient effector T cells accumulated to a greater extent than wild type T cells, and preferentially generated memory precursor cells that displayed enhanced accumulation and functional capacity in response to TCR stimulation as persisting memory cells. Although, PSGL-1-deficient memory cells did not exhibit inherent greater sensitivity to cell death, they failed to respond to a homologous virus challenge after adoptive transfer into naïve hosts indicating an impaired capacity to generate memory effector T cell responses in the context of viral infection. These studies underscore the function of PSGL-1 as a key negative regulator of effector and memory T cell differentiation and suggest that PSGL-1 may limit excessive stimulation of memory T cells during acute viral infection.

## Introduction

Immunological memory confers host protection through an immune response that is more robust and effective at neutralizing a previously encountered pathogen. T cell-mediated immunity to virus infections requires differentiation of effector T cells that contribute to pathogen clearance by interfering with viral replication and by direct killing of virus-infected cells. As the infection is resolved memory T cells are generated that promote long-term host protection against re-infection (1). The signals determining the generation of memory T cells remain incompletely understood, and depend upon contextual cues that include the duration of antigen exposure, the degree of inflammation, as well as the tissue localization and distribution of infection. Both CD8^+^ and CD4^+^ T cells can be classified as central memory (T_CM_) and effector memory (T_EM_) cells that are broadly distinguished by their preferential migration through, and responses in lymphoid and non-lymphoid tissues, respectively. An additional subset, designated tissue resident memory cells (T_RM_), that are retained at the sight of initial infection where they locally provide protective immunity (2). It is the integration of a multitude of signals in diverse microenvironments that regulates the transcriptional and epigenetic programming, which determines effector and memory T cell fates (3). The fine-tuning of T cell responses to viral pathogens is in part achieved by appropriate engagement of negative regulatory molecules to prevent excessive T cell responses (4). It is now apparent that signals through PD-1 regulate effector CD8^+^ T cell responses (5) and both PD-1 and LAG-3 impact the generation and responses of memory T cells in acute viral infections (6–8). We previously identified that the adhesion receptor, P-selectin glycoprotein-1 (PSGL-1), is a key T cell-intrinsic inhibitory receptor that is required for the development of T cell exhaustion in chronic virus infection and determines the expression levels of multiple inhibitory receptors that include PD-1, TIM-3, and LAG-3 among others (9). We now address whether PSGL-1 can also play a fundamental role in the regulation of effector and memory T cell differentiation and responses.

PSGL-1 (encoded by the gene *Selplg*) is expressed on the surface of most hematopoietic cells and well-known for its role in regulating leukocyte migration into sites of infection *via* binding of P-selectin on inflamed vascular endothelium. Selectin-binding requires post-translational modifications that include sulfation and glycosylation, which are constitutively present on PSGL-1 expressed by innate immune cells (10). Naïve T cells lack these modifications and are thus unable to bind selectins, but can acquire selectin binding capacity during effector differentiation (11), although expression is typically transient. On T cells, however, PSGL-1 can engage additional ligands in the absence of, or independently of selectin binding (12). These include the lymphoid tissue chemokines, CCL19 and CCL21 (13), which regulate the entry and positioning of T cells and dendritic cells in secondary lymphoid organs (14), and VISTA (V-type immunoglobulin domain-containing suppressor of T cell activation) (15), a negative regulator of T cell responses, which is primarily expressed on myeloid cells (16), respectively. The role of PSGL-1 as a potential negative regulator of T cells was initially identified in studies showing that PSGL-1-deficient CD8^+^ T cells displayed greater homeostatic turnover in the absence of overt activation, as well as enhanced persistence as memory cells after acute infection with LCMV Armstrong (Arm) strain (17). However, whether PSGL-1-deficiency cells alters T cell function or differentiation, or secondary responses has not been addressed.

Here we report that PSGL-1 is a negative regulator of T cell responses to LCMV Arm infection that limits the responses of effector and memory T cells. With primary infection, virus-specific CD8^+^ and CD4^+^ *Selplg^-/-^* T cells accumulated to a much greater extent than wild type (WT) cells and displayed greater persistence as memory T cells due to better intrinsic survival. As effectors, *Selplg^-/-^* T cells expressed increased levels of the receptors, IL-7Rα and IL-2Rβ, as well as lower levels of PD-1 and developed a predominantly memory precursor/progenitor phenotype when compared to WT T cells. We observed greater numbers of cytokine-producing virus-specific CD4^+^ and CD8^+^ T cells in *Selplg^-/-^* mice at both the effector and memory stages after infection. In sharp contrast, however, although *Selplg^-/-^*memory T cells responded effectively to restimulation by LCMV viral peptides *in vitro* and did not exhibit greater susceptibility to death in the context of TCR engagement *in vivo*, they failed to respond to a secondary challenge with LCMV Arm after adoptive transfer with WT cells into the same naïve host, thus suggesting that inhibition *via* PSGL-1 during memory T cell activation may limit excessive stimulation induced by viral infection. These studies underscore that PSGL-1 has a fundamental role as a regulator of effector and memory T cell differentiation and indicate that PSGL-1-dependent inhibition may be essential for memory effector anti-viral responses.

## Materials and Methods

### Mice

C57BL/6J and *Selplg^-/-^* mice (18) were purchased from Jackson Laboratory and then bred in specific-pathogen-free (SPF) facilities and maintained in biosafety level 2 (BSL-2) facilities after infection in the vivaria at SBP and UC Irvine. *Selplg^-/-^* mice were backcrossed to C57BL/6J mice for more than ten generations. P14 and SMARTA mice were obtained from The Scripps Research Institute (originally from Dr. Charles D. Surh). These mice were bred to Ly5.1 (B6.SJL-Ptprc^a^ Pepc^b^/BoyJ) mice and to Thy1.1 (B6.PL-Thy 1^a^/CyJ) *Selplg^-/-^* mice which were purchased from Jackson Laboratory and bred in house. Both male and female mice were used and were greater than 6 weeks of age. All experiments were approved by the animal care and use committees at SBP (A3053-01) and UC Irvine (AUP-18-148).

### Virus Infection and Titers

LCMV Armstrong (Arm) strain was propagated in baby-hamster kidney cells and titrated on Vero African-green-monkey kidney cells (19, 20). Frozen stocks were diluted in Vero cell media and mice were infected by intraperitoneal (i.p.) injection of 2 x 10^5^ plaque-forming units (PFUs) of LCMV Arm. Virus titers were determined from serial dilutions of sera taken from mice at 4 days post infection (dpi) using a focus forming assay (21).

### Adoptive Transfer

Naïve P14 WT or *Selplg* ^-/-^ T cells or SMARTA WT or *Selplg* ^-/-^ T cells were isolated from the spleens by magnetic sorting (Stem Cell Technologies, negative selection) according to the manufacturers protocol. WT and *Selplg* ^-/-^ P14 or SMARTA cells were transferred in equal numbers (1000 each) into C57BL/6 mice by i.v. injection. On the same day, the mice were infected with LCMV Arm by i.p. injection. For memory cell adoptive transfer, viable WT and *Selplg* ^-/-^ P14 cells were isolated from the spleens of mice at 30dpi by FACS sorting based on the allelic markers. WT and *Selplg* ^-/-^ P14 memory cells were coinjected into naïve C57BL/6 recipients in a dose of 2000 each by i.v. injection, followed by i.p. infection with LCMV Arm.

### Flow Cytometry

Cells from the spleens or pooled lymph nodes (inguinal, axial, brachial) were dissociated in HBSS. For cell surface staining, 2x10^6^ cells were incubated with antibodies in staining buffer (PBS, 2% fetal bovine serum (FBS) and 0.01% NaN_3_) for 20 minutes at 4°C and with H-2D^b^-GP_33-41_, H-2D^b^-GP_276-286_, H-2D^b^-NP_396-404_, or IA^b^-_66-77_ tetramers (NIH core facility) for 1 hour and 5 minutes at room temperature. For functional assays, cells from infected animals were cultured for 5 hours at 37°C with 2ug/mL of GP_33-41_, GP_276-286_, NP_396-404_, and GP_61-80_ peptides (AnaSpec) in the presence of brefeldin A (1μg/ml; Sigma-Aldrich). The cells were then stained with antibodies for expression of surface proteins, fixed, permeabilized, and stained with antibodies for intracellular cytokine detection. To evaluate cell degranulation, splenocytes were incubated in the presence of anti-CD107a-FITC (Biolegend). The culture media was RPMI-1640 containing 10 mM HEPES, 1% non-essential amino acids and L-glutamine, 1mM sodium pyruvate, 10% heat-inactivated FBS, and penicillin/streptomycin antibiotics. The following anti-mouse antibodies were used in this study: Biolegend CD4 (RM4-5), CD8 (53-6.7), CD62L (MEL-14), CD127(A7R34), CD122 (TM-β1), CD107a (1D4B), IFN-γ (XMG1.2), TNF-α (MP6-XT22), CD11a (M17/4), CD49d (R1-2), CD45.1 (A20), CD90.1 (OX-7), KLRG-1 (2F1/KLRG1), PD-1 (29F.1A12), CD95 (Fas, SA367H8), CD178 (FasL, MFC3). Caspase-3 staining was done using CaspGLOW Fluorescein Active Caspase-3 staining kit (ThermoFisher) and following manufacturer’s instructions.

### *In Vivo* Proliferation

Mice were injected i.p. with 2 mg BrdU (Sigma-Aldrich) 16 hours before removing the spleens at 9dpi to measure proliferation by flow cytometry after intracellular staining with an anti-BrdU antibody. BrdU staining was done using a BrdU Flow kit (BD Biosciences) following the manufacturer’s instructions. Cells were acquired with a Novocyte3000 flow cytometer.

### Data Analysis

Flow cytometry data were analyzed with FlowJo software (TreeStar). Graphs were prepared with GraphPad Prism software. GraphPad Prism was used for statistical analysis to compare outcomes using a two-tailed unpaired Student’s t test; significance was set to p <0.05 and represented as *<0.05, **<0.005, ***<0.001, and ****<0.0001. Error bars show SEM.

## Results

### *Selplg^-/-^* Mice Infected With LCMV Have an Increased Accumulation of Effector and Memory T Cells

**Figure 1:**
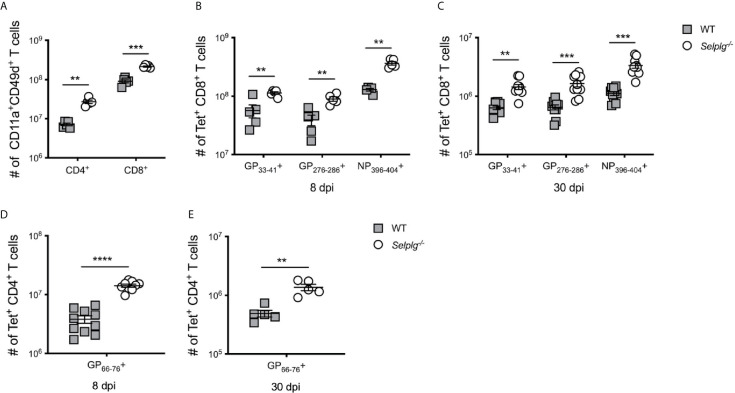
Increased accumulation of *Selplg-/-* T cells during LCMV infection. WT and *Selplg^-/-^* mice were infected with LCMV Armstrong by i.p. injection. **(A)** The numbers of antigen responding CD11a^+^CD49d^+^ T cells in spleen at 8dpi. Tetramer^+^ CD8^+^ T cells in the spleens were enumerated at 8dpi **(B)** and 30dpi **(C)**. Tetramer^+^ CD4^+^ T cells were enumerated in the spleens at 8dpi **(D)** and 30dpi **(E)**. Data are representative of four independent experiments (n = 5 or more mice/group). Graphs show the mean ± SEM. **p < 0.005, ***p < 0.001, ****p < 0.0001 by two-tailed unpaired *t-*test.

To investigate whether PSGL-1 expression regulated T cell responses to primary virus infection, we infected WT and *Selplg* ^-/-^ mice with LCMV Arm. We first quantified virus-specific T cells in the spleen as indicated by co-expression of CD11a^+^CD49d^+^ (22) and found increased numbers of effector CD4^+^ and CD8^+^ T cells at 8dpi, indicating a larger population of T cells was responding to the virus (**Figure 1A**). We next quantified the numbers of virus-specific CD8^+^ T cells in the spleens by staining with the MHC-I tetramers specific for GP_33-41_, GP_276-286_, and NP_396-404_ LCMV epitopes recognized by CD8^+^ T cells with differing TCR affinities (23). We also observed significant increases in the numbers of virus epitope-specific CD8^+^ T cells in *Selplg^-/-^* mice at 8dpi (**Figure 1B**) and importantly, these differences were maintained at 30dpi (**Figure 1C**) long after viral clearance, which occurs by 8dpi. However, the frequencies of virus-specific CD8^+^ T cells were selectively increased in ${\text{NP}}_{396-404}^{+}$ cells from *Selplg* ^-/-^ mice compared to WT mice (**Figures S1A, B**), suggesting a more pronounced effect of PSGL-1-deficiency on the highest affinity LCMV-specific CD8^+^ T cell clone (23). We next investigated whether CD4^+^ T cell responses were different in *Selplg^-/-^* mice compared to WT mice and found that, like CD8^+^ T cells, the numbers of virus-specific CD4^+^ T cells were significantly increased at 8dpi (**Figure 1D**) as well as at 30dpi (**Figure 1E**) in the spleen with *Selplg*-deficiency as were the frequencies (**Figures S1C, D**). Furthermore, longitudinal analyses revealed that the differences in the recovery of virus-specific CD8^+^ and CD4^+^ T cells in the spleens of *Selplg^-/-^* mice were detectable as early as 5dpi and sustained thereafter (**Figures S2A, B**). We found comparable results in the lymph nodes (**Figures S2C, D**), indicating that impaired trafficking into these sites with *Selplg*-deficiency did not account for the differences in recovery that were observed. Together, these findings show that with *Selplg-*deficiency, both virus-specific CD4^+^ and CD8^+^ T cells exhibit an enhanced response to acute LCMV infection as indicated by the significant increases in the numbers that accumulated during the effector response, differences that were maintained in the memory stage after viral clearance.

### *Selplg^-/-^* CD8^+^ T Cells Exhibit Enhanced *S*urvival but Not Increased Proliferation

**Figure 2:**
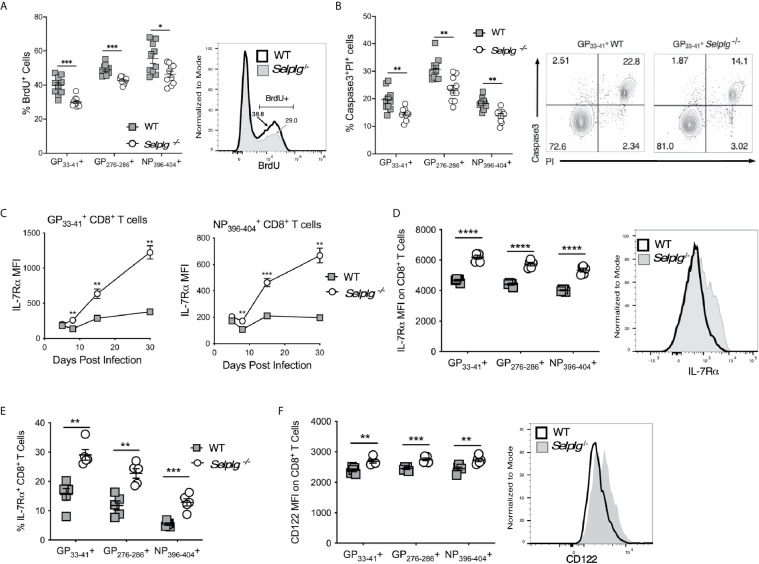
*Selplg^-/-^* effector and memory T cells have increased survival. WT and *Selplg^-/-^* mice were infected with LCMV Armstrong by i.p. injection. **(A)** CD8^+^ T cells were analyzed for the frequencies of BrdU incorporating CD8^+^ tetramer^+^ T cells, with a representative histogram for $\text{G}{\text{P}}_{33-41^{+}}$ cells. **(B)** Frequencies of Caspase 3^+^PI^+^ CD8^+^ tetramer^+^ T cells at 8dpi, with a representative dot plot showing the frequencies for $\text{G}{\text{P}}_{33-41^{+}}$ T cells. **(C)** Geometric mean fluorescence intensity (MFI) of IL-7Rα levels expressed on CD8^+^ tetramer^+^ cells from the blood at the indicated time points and **(D)** at 30dpi from the spleen, with a representative histogram for $\text{G}{\text{P}}_{33-41^{+}}$ cells. **(E)** The frequencies of IL-7Rα^+^, tetramer^+^ CD8^+^ T cells. **(F)** The levels CD122 expressed on tetramer^+^ cells in spleen at 30dpi expressed as MFI, with a representative histogram for $\text{G}{\text{P}}_{33-41^{+}}$ cells. Data are representative of three independent experiments (n = 5 or more mice/group). Graphs show the mean ± SEM. *p < 0.05, **p < 0.005, ***p < 0.001, ****p < 0.0001 by two-tailed unpaired *t-*test **(A–F)**.

To address whether the increased recovery of virus-specific T cells in *Selplg^-/-^* mice was a result of differences in their proliferation and/or survival, we infected WT and *Selplg^-/-^* mice and injected BrdU 16 hours prior to analysis at 8dpi. BrdU incorporation by CD8^+^ T cells *in vivo* showed that both WT and *Selplg^-/-^* CD8^+^ tetramer^+^ cells proliferated; however, *Selplg^-/-^* CD8^+^ T cells had decreased frequencies of BrdU^+^ cells compared to WT at 8dpi (**Figure 2A**). Since we did not observe enhanced proliferation in *Selplg^-/-^* T cells, we next analyzed apoptosis by evaluating virus-specific CD8^+^ T cells for their levels of active caspase 3 and propidium iodide (PI) uptake, and found that compared to WT cells, *Selplg^-/-^* CD8^+^ T cells had lower frequencies of caspase 3^+^PI^+^ cells, indicating that these cells had decreased apoptosis (**Figure 2B**).

Since cytokines such as the γc cytokines, IL-2, IL-7 and IL-15, can promote CD8^+^ T cell survival and can impact the differentiation of effector and memory T cells (24), we examined whether differences in expression of IL-7Rα or CD122 (IL-2Rβ), which is part of both the IL-2 and IL-15 receptors, distinguished WT and *Selplg^-/-^* CD8^+^ T cells. We found dramatic increases IL-7Rα expression on *Selplg^-/-^* CD8^+^ T cells by 8dpi and higher expression compared to WT cells up to 30dpi (**Figure 2C**). We also found increased expression of IL-7 Ra (**Figure 2D**) and frequencies of IL-7Rα^+^ virus-specific CD8^+^ T cells (**Figure 2E**) with all 3 tetramer^+^ populations, shown at 8dpi. Increases in CD122 expression on *Selplg^-/-^* memory CD8^+^ T cells were also found with all three CD8^+^ tetramer^+^ populations (**Figure 2F**), shown at 30dp**i**. These findings indicate that PSGL-1 expression can reduce the expansion of anti-viral effector T cells by limiting their survival, to which lower levels of cytokine receptor expression could contribute.

### Virus-Specific *Selplg^-/-^* CD8^+^ T Cells Become Enriched in Memory Precursor and Central Memory Phenotype Cells

**Figure 3:**
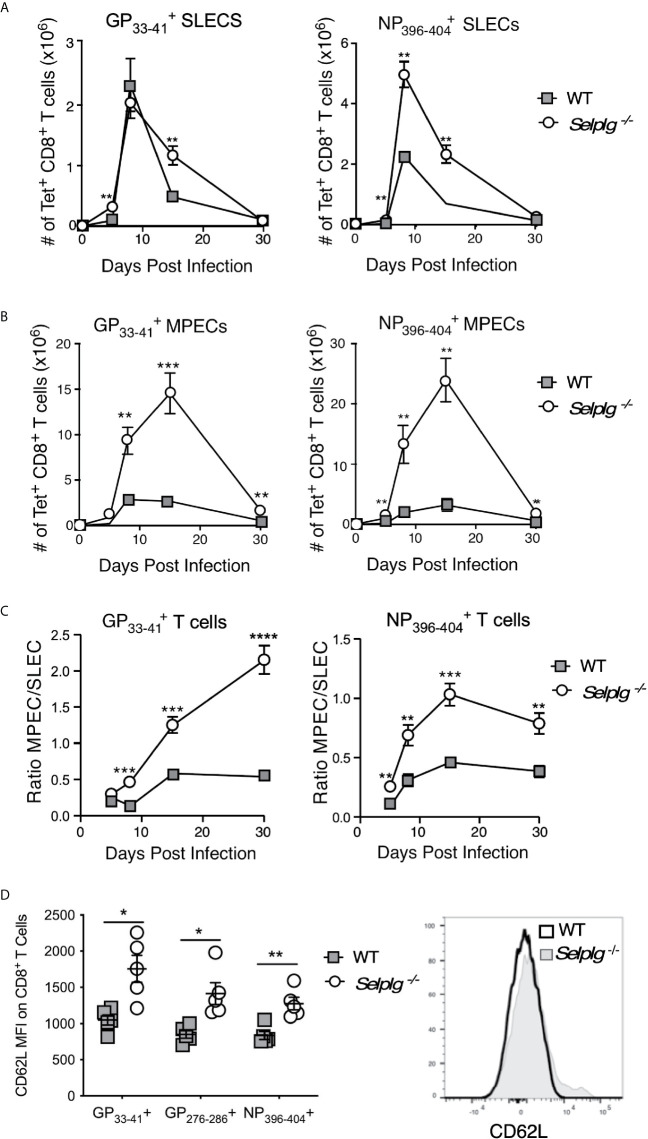
Virus-specific CD8^+^ T cells in *Selplg^-/-^* mice are enriched for memory precursor and central memory phenotype cells. WT and *Selplg^-/-^* mice were infected with LCMV Arm and the numbers of short-lived effector phenotype cells (SLECs: IL7Rα^-^, KLRG-1^+^) **(A)** and memory precursor effector cells (MPECs, IL-7Rα^+^, KLRG-1^-^) **(B)** were evaluated for $\text{G}{\text{P}}_{33-41^{+}}$ CD8^+^ T cells and $\text{N}{\text{P}}_{396-404^{+}}$ CD8^+^ T cells in the spleens. **(C)** The ratios of MPEC to SLEC phenotype CD8^+^ T cells for each of the tetramer+ populations. **(D)** Expression levels (MFI) of CD62L on CD8^+^ tetramer^+^ T cells from the spleens at 30dpi, with a representative histogram for $\text{G}{\text{P}}_{33-41^{+}}$ CD8^+^ T cells. Data are representative of three independent experiments (n = 5 or more mice/group). Graphs show the mean ± SEM *p < 0.05, **p < 0.005, ***p < 0.001, ****p < 0.0001 by two-tailed *t-*test.

The increased levels of IL-7Rα on virus-specific CD8^+^ T cells from *Selplg* ^-/-^ mice compared to WT mice suggested the possibility of altered differentiation of CD8^+^ T cells during the course of viral infection. Thus, we analyzed virus-specific CD8^+^ T cell subsets by examining the reciprocal expression of IL-7Rα and KLRG-1 which distinguishes memory precursor effectors (MPECs: IL-7Rα^+^, KLRG-1^−^) from more terminally differentiated and more short-lived effectors (SLECs: IL-7Rα^−^, KLRG-1^+^) in the LCMV Arm model (25). *Selplg^-/-^* mice had an increased accumulation of SLECs as early as 5dpi in both ${\text{NP}}_{396-404}^{+}$ and ${\text{GP}}_{33-41}^{+}$ CD8^+^ T cells (**Figure 3A**). These differences were also evident at later stages of the response (15dpi). A similar pattern was observed with respect to accumulation of virus-specific *Selplg^-/-^* CD8^+^ T cells that bore a MPEC phenotype (**Figure 3B**), although both ${\text{NP}}_{396-404}^{+}$ and ${\text{GP}}_{33-}^{41}+$ CD8+ T cells showed significantly greater recovery throughout the effector response and after memory formation in *Selplg^-/-^* mice. Moreover, comparison of the ratios of MPEC to SLEC phenotype cells showed that memory precursors comprised a much greater proportion of virus-specific CD8^+^ T cells in *Selplg^-/-^* mice (**Figure 3C**). Not only did memory T cells from these mice express higher levels of IL-7Rα than WT cells (**Figures 2C, D**), we also observed higher expression of CD62L on the memory population as a whole, indicating greater representation of T_CM_ phenotype cells (**Figure 3D**). The results show that PSGL-1 expression limited the development of both SLECs and MPECs to regulate the size of the memory T cell pool, but also suggest that the absence of PSGL-1-dependent inhibition could alter the differentiation of CD8^+^ T cells towards a MPEC phenotype or may promote a loss of SLECs due to terminal differentiation. Greater representation of T_CM_ cells, which are also considered to be memory progenitors, supports the concept that CD8^+^ T cells with greater memory potential are generated in the context of PSGL-1-deficiency after LCMV Arm infection.

### Increased Functional Anti-Viral T Cells Are Generated in *Selplg^-/-^* Mice

**Figure 4:**
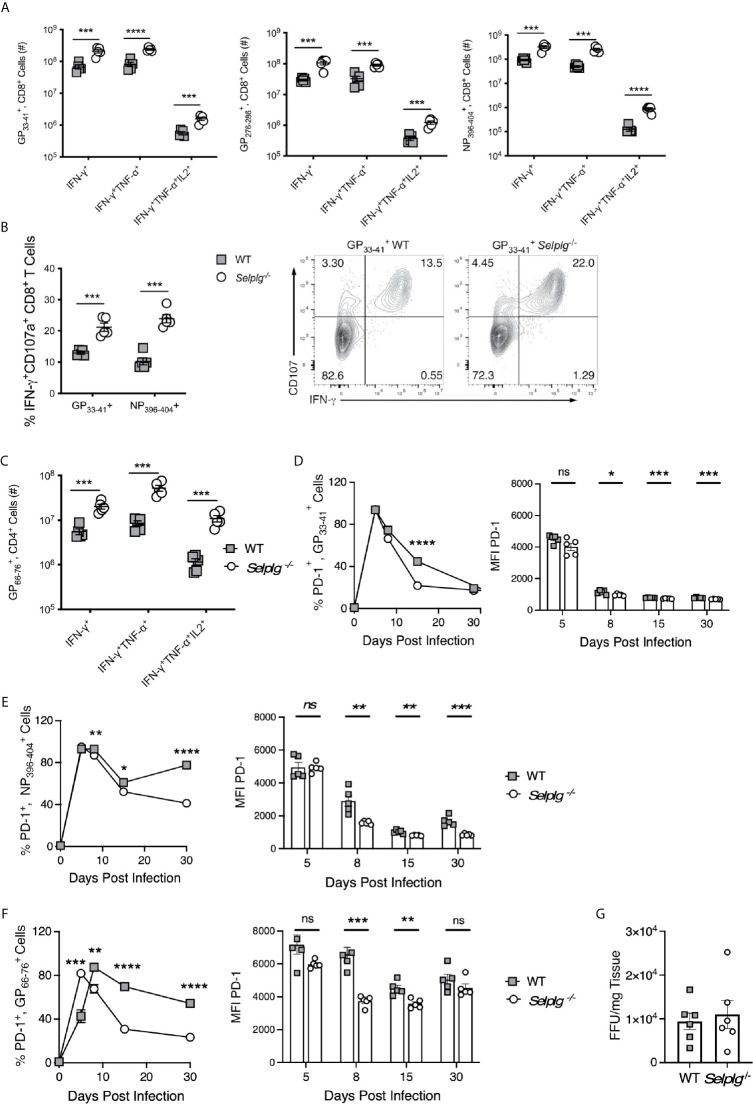
Virus-specific T cells in *Selplg^-/-^* mice have increased effector function and altered PD-1 expression. WT and *Selplg^-/-^* mice were infected with LCMV Arm and their spleens isolated at 8dpi. Splenocytes were stimulated *ex vivo* with the indicated LCMV peptides and assessed for the **(A)** and the numbers of cytokine^+^ CD8^+^ T cells **(B)** and the frequencies of IFN-γ^+^CD107a^+^ CD8^+^ T cells, **(C)** in addition to the numbers of cytokine^+^ CD4^+^ T cells. **(D, E)** % PD-1^+^ tetramer^+^, CD8^+^ T cells and expression of PD-1(MFI). **(F)** % PD-1^+^ tetramer^+^, CD4^+^ T cells and expression of PD-1 (MFI). **(G)** Virus titers measured from the spleens at 4dpi by focus forming units (FFU) and expressed/g of tissue. Data in **(A–F)** are representative of three independent experiments (n = 5 or more mice/group). Data in **(G)** are from one experiment. Graphs show the mean ± SEM *p < 0.05, **p < 0.005, ***p < 0.001, ****p < 0.0001 by two-tailed unpaired *t-*test.

We next evaluated whether functional differences existed between WT and *Selplg^-/-^* T cells in response to LCMV Arm infection. We thus examined cytokine production of anti-viral T cells *via ex vivo* peptide stimulation and found that while both WT and *Selplg^-/-^* T cells produced effector cytokines, significantly greater numbers of virus-specific CD8^+^ T cells producing IFN-γ, IFN-γ and TNF-α, and IFN-γ, TNF-α, and IL-2 were detected in *Selplg^-/-^* mice at 8dpi (**Figure 4A**). Greater frequencies were also observed for ${\text{NP}}_{396-404}^{+}$ cells (**Figure S3A**). In addition, we detected increased frequencies of CD107^+^IFN-γ^+^ CD8^+^ T cells in *Selplg^-/-^* mice, indicating that they had increased degranulation capacity, which reflects cytotoxic activity (**Figure 4B**). Consistent with the increase in cytokine-producing T cells in *Selplg^-/-^* mice, we also observed increased numbers (**Figure 4C**) and frequencies (**Figure S3B**) of cytokine^+^ CD4^+^ T cells. There were significant increases in IFN-γ^+^, IFN-γ^+^TNF-α^+^, and IFN-γ^+^TNF-α^+^IL-2^+^ cells in *Selplg^-/-^* mice and importantly, we detected more virus-specific CD4^+^ and CD8^+^ T cells that produced all three cytokines (**Figures 4A, C**). These studies showed that both WT and *Selplg^-/-^* T cells differentiated into functional effector T cells, but that that greater numbers and frequencies of polyfunctional T cells were generated in *Selplg^-/-^* mice.

We previously found that *Selplg^-/-^* CD8^+^ T cells generated during chronic LCMV infection had reduced expression of PD-1 as well as other inhibitory receptors (9). Since PD-1 is upregulated on effector CD8^+^ T cells responding to LCMV Arm (5), we examined whether *Selplg* ^-/-^ virus-specific CD8^+^ T cells expressed differences in PD-1 levels during acute infection. We found comparable high frequencies of PD-1^+^ tetramer^+^ CD8^+^ T cells in WT and *Selplg^-/-^* mice at 5dpi, and these cells initially upregulated PD-1 expression to similar levels as WT cells. However, by 8dpi, the frequencies of PD-1^+^ tetramer^+^ CD8^+^ T cells in both groups of mice began to drop, with dramatically decreased expression levels on *Selplg*-^/-^ tetramer^+^ CD8^+^ T compared to their WT counterparts (**Figures 4D, E**). Greater frequencies of PD-1^+^ cells were sustained in both WT and *Selplg^-/-^* mice, particularly in the NP_396-404_ tetramer^+^ population up to 30dpi, the length of our analysis in this experiment. Similar outcomes were observed with ${\text{GP}}_{66-76}^{+}$. CD4^+^ T cells (**Figure 4F**). However, by 48dpi, PD-1 expression was lost on WT as well as *Selplg^-/-^* T cells (not shown). Together the data suggest that PSGL-1 impacts PD-1 levels on virus-specific T cells particularly during the transition to memory cells, which may indicate differences in activation status by WT compared to *Selplg* ^-/-^ T cells. Overall our results suggest that lower expression of PD-1 with PSGL-1-deficiency could contribute to the greater representation of MPECs and T_CM_ cells at the memory phase (**Figure 3**). Notably, we did not detect differences in expression of other inhibitory receptors (LAG-3 or TIM-3, not shown).

Since greater T cell functionality accompanied PSGL-1 deficiency, we considered the possibility that a more effective response could reduce viral loads, thereby hastening the effector to memory transition and limiting the differentiation of SLECs. Thus, we measured viral load at 4dpi (5) when LCMV Arm is detectable in the spleen although not in the blood. We did not find differences in viral titers at this time (**Figure 4G**), although the virus levels were variable. The results suggest that capacity for early viral containment is not affected by the subsequent greater accumulation of effector T cells with PSGL-1-deficiency.

### Increased Functionality of Memory Anti-Viral T Cells in *Selplg^-/-^* Mice

**Figure 5:**
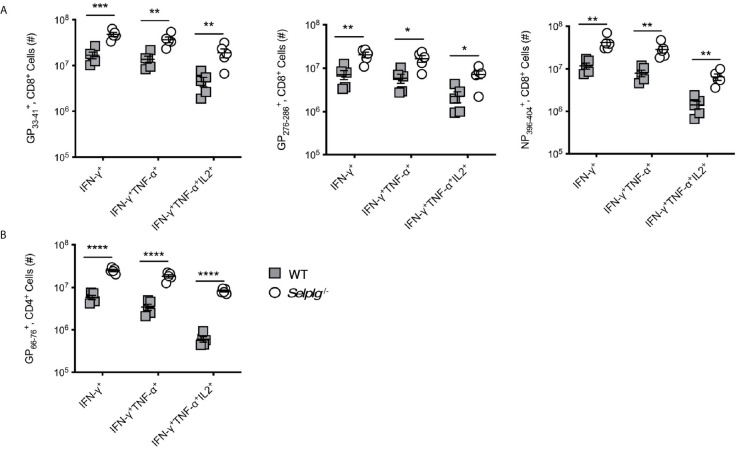
*Selplg^-/-^* mice have increased accumulation of cytokine producing memory T cells. WT and *Selplg^-/-^* mice were infected with LCMV Armstrong and the spleens were isolated at 30dpi. Splenocytes were stimulated with the indicated viral peptides and the numbers of cytokine^+^ CD8^+^ T cells **(A)** and CD4^+^ T cells **(B)** were enumerated. Data are representative of three independent experiments (n = 5 or more mice/group). Graphs show the mean ± SEM *p < 0.05, **p < 0.005, ***p < 0.001, ****p < 0.0001 by two-tailed unpaired *t-*test.

We next assessed the functional capacity of virus-specific memory T cells from WT and *Selplg^-/-^* mice as measured by cytokine production. As observed at 8dpi, at 30dpi we detected increased numbers of CD8^+^ T cells in *Selplg^-/-^* mice that were IFN-γ^+^, IFN-γ^+^, TNF-α^+^, and IFN-γ^+^TNF-α^+^IL-2^+^ after restimulation with viral peptides (**Figure 5A**). The *Selplg^-/-^* CD4^+^ T cell population also contained more IFN-γ^+^, IFN-γ^+^TNF-α^+^, and IFN-γ^+^TNF-α^+^IL-2^+^ producers at 30dpi (**Figure 5B**). Furthermore, we observed increased frequencies and numbers of cytokine^+^CD8^+^ T cells (**Figures S4A, B**) and CD4^+^ T cells (**Figure S4C**). Together, our findings indicate that with LCMV Arm infection, functional *Selplg^-/-^* memory T cells persist in greater numbers, and that a greater fraction produce effector cytokines after restimulation with viral antigens.

### The Accumulation of *Selplg^-/-^* T Cells During Viral Infection Is Cell-Intrinsic

**Figure 6:**
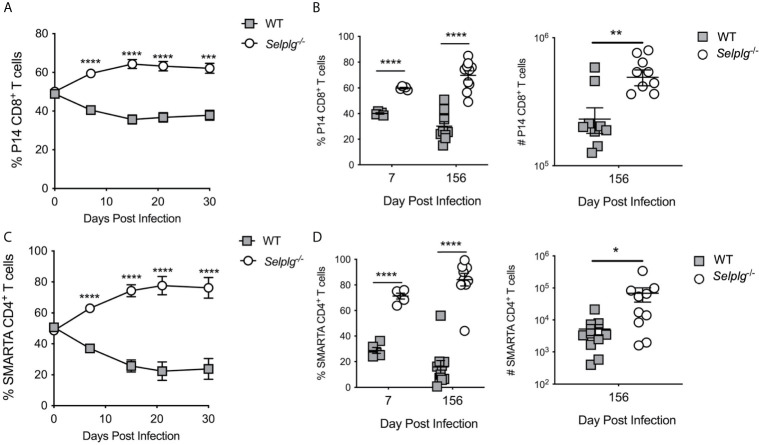
*Selplg^-/-^* T cells display increased cell-intrinsic accumulation as effector and memory T cells. **(A)** Frequencies of WT and *Selplg* ^-/-^ P14 T cells in the blood after adoptive transfer into naïve WT recipients and infection with LCMV Arm. **(B)** Frequencies and numbers of P14 T cells in the spleens at 7- and 156dpi. **(C)** Frequencies of WT and *Selplg* ^-/-^ SMARTA T cells in the blood after adoptive transfer and infection with LCMV Arm. **(D)** Frequencies and numbers of SMARTA T cells in the spleens at 7- and 156dpi. Data are representative of three independent experiments (n = 5 or more mice/group). Graphs show the mean ± SEM *p < 0.05, **p < 0.005, ***p < 0.001, ****p < 0.0001 by two-tailed unpaired *t-*test.

Since *Selplg^-/-^* mice mounted a more robust CD8^+^ T cell response during acute LCMV infection, we next determined whether this accumulation was cell-intrinsic. We co-transferred WT and *Selplg* ^-/-^ TCR transgenic (P14) CD8^+^ T cells at a 1:1 ratio in WT mice and examined their expansion after LCMV Arm infection. We found that *Selplg^-/-^* P14 T cell frequencies were increased in the blood from 7dpi through 30dpi (**Figure 6A**) during which time a ~1.5 ratio of knockout to WT cells within P14 cells was maintained. However, both WT and *Selplg* ^-/-^ P14 cells decayed over time (**Figure S5**). To address whether the survival differences were further sustained, we compared the frequencies of P14 cells in the blood at 7dpi and 156dpi (**Figure 6B**, left panel) as well as the numbers in the spleens 156dpi (**Figure 6B**, right panel). The results demonstrate that consistently greater recovery of *Selplg* ^-/-^ P14 cells compared to their WT counterparts. We next co-transferred WT and *Selplg^-/-^* T cell receptor transgenic CD4^+^ (SMARTA) T cells at a 1:1 ratio into naive WT mice that were subsequently infected with LCMV Arm. We observed significantly greater recovery of *Selplg* ^-/-^ SMARTA cells compared to WT SMARTA cells in the blood from 7dpi to 30dpi, which stabilized at a ~3 fold difference by 15dpi (**Figure 6C**). Both populations decayed with time (**Figure S5**). We also found that increased frequencies of *Selplg^-/-^* SMARTA^+^ T cells were sustained at 156dpi in the blood, at which time increased numbers of *Selplg^-/-^* SMARTA T cells in the spleens were also observed (**Figure 6D**). Our findings show that the greater persistence of *Selplg^-/-^* virus-specific CD8^+^ and CD4^+^ T cells is cell-intrinsic, and that PSGL-1-deficiency increased the numbers of memory T cells that were maintained long-term.

### Greater MPEC Representation Does Not Support *Selplg* ^-/-^ Memory T Cell Responses After Secondary Viral Challenge

**Figure 7:**
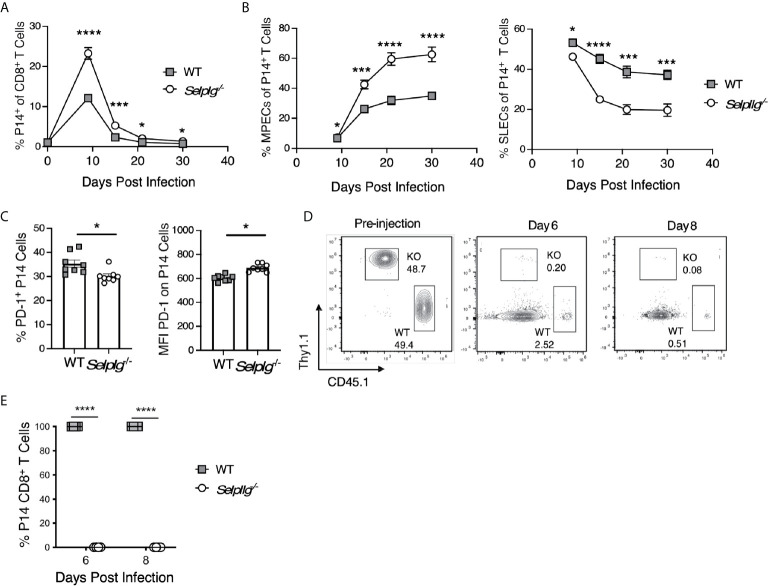
Greater MPEC representation does not support *Selplg* ^-/-^ memory T cell responses after secondary viral challenge. WT and *Selplg^-/-^* memory P14 CD8^+^ T cells were isolated from the spleens of WT mice at 30 days after cotransfer in a 1:1 ratio and infection with LCMV Armstrong. These cells were then re-injected in equivalent numbers into WT naïve hosts and mice that were then infected with LCMV Armstrong. **(A)** The frequencies of WT and *Selplg* ^-/-^ P14 T cells in the blood with time. **(B)** The frequencies of the memory precursor effector cells (MPECs, IL-7Rα^+^, KLRG-1^-^) and short-lived effector phenotype cells (SLECs: IL7Rα^-^, KLRG-1^+^) as a % of WT and *Selplg* ^-/-^ P14 cells. **(C)** Frequencies PD-1^+^ cells and their and expression levels of PD-1 (MFI). **(D)** representative flow cytometry plots for the pre-injection populations of P14 cells, and **(E)** their recovery at 6 and 8 dpi **(B)**. Data are representative of three independent experiments (n = 5 or more mice/group). Graphs show the mean ± SEM *p < 0.05, ***p < 0.001, ****p < 0.0001 by two-tailed unpaired *t-*test.

Since we observed increased representation of memory precursor/progenitor CD8^+^ T cells after primary LCMV infection in *Selplg* ^-/-^ mice, we assessed their whether their altered differentiation was T cell intrinsic. Thus, we followed the recovery of adoptively co-transferred WT and *Selplg* ^-/-^ P14 cells in the blood with time after infection and found maintenance of ~2 fold higher *Selplg* ^-/-^ cells compared to WT cells (**Figure 7A**). *Selplg^-^* ^/-^ P14 cells displayed greater recovery of MPECs and decreased recovery of SLECs compared to WT P14 cells (**Figure 7B**). At 30dpi, we found PD-1^+^ cells both the WT and *Selplg^-^* ^/-^ P14 populations, although PD-1 expression by these cells was at low levels (**Figure 7C**). We next assessed the impact of PSGL-1-deficiency on the response of memory P14 cells to viral challenge. In order to avoid the confounding T cell memory response of the initial host, differences in the frequencies of virus-specific T cells and the existence of LCMV-specific antibodies, we sorted WT and *Selplg^-/-^* P14^+^ T cells from WT mice at 30dpi after they had that received both populations in a 1:1 ratio at the time of infection. We co-injected these memory populations in equal numbers into naive WT hosts that were then infected with LCMV Arm. We detected robust expansion/recovery of WT P14 T cells at 6dpi, with contraction occurring by 8dpi in the spleens. However, few *Selplg^-/-^* P14 T cells were recovered (**Figures 7D, E**). *Selplg* ^-/-^ memory T cells were also not detected at 4- or 6dpi in in the peripheral lymph nodes, mesenteric lymph nodes, Peyer’s patches, lungs or liver (not shown). These data indicate that redistribution of P14 memory T cells is not likely to account for their lack of accumulation upon viral challenge.

To address the potential for preferential cell death by *Selplg* ^-/-^ P14 cells, we assessed the impact of TCR restimulation *in vivo* by injecting GP_33-41_ peptide together with LPS as an adjuvant. As observed previously, *Selplg* ^-/-^ P14 cells were recovered in greater frequencies than WT P14 cells at 30dpi. However, there were no differences in the frequencies of Caspase 3^+^ cells (**Figure S6B**), or of CD95^+^ and CD95L^+^ cells (**Figure S6C**) after TCR stimulation *in vivo*. These findings suggest that TCR-induced cell death or death receptor-mediated cell death does not account for the disappearance of PSGL-1-deficient memory T cells. The data support the possibility that PSGL-1 expression by memory T cells is required to limit memory effector CD8^+^ T cell stimulation and responses, thereby sustaining effector activity in the context of the extensive inflammatory milieu that develops with acute LCMV infection.

## Discussion

In this study we demonstrated that PSGL-1, in its function as a T cell-intrinsic inhibitory receptor, limits the magnitude of CD4^+^ and CD8^+^ T cell responses to acute infection with LCMV Arm, and correspondingly constrains the size of the memory cell pool. Accordingly, *Selplg^-/-^* mice had notable increases in the numbers of virus-specific CD4^+^ and CD8^+^ T cells following acute LCMV infection that were evident by 5dpi as well as at the peak of the effector T cell response (8dpi), and they maintained increased numbers of persisting memory T cells (≥30dpi). Phenotypic analyses of CD8^+^ T cells indicated that there were greater numbers of effector cells in both the SLEC and MPEC populations in *Selplg* ^-/-^ mice compared to WT mice, but that the representation of MPECs compared to SLECs was significantly increased. Previous studies showed that dendritic cells from *Selplg* ^-/-^ mice have a greater capacity to stimulate naïve T cells *in vitro* (26), which suggests that changes P innate immune cells could also impact the magnitude of T cell responses in the context of germ-line deficiency of PSGL-1. However CD8^+^ T cells displayed intrinsic greater generation of MPECs, supporting a conclusion that PSGL-1-deficiency favors the differentiation of cells with memory progenitor potential. This conclusion is supported by our finding of increased representation of CD8^+^ T_CM_ compared to T_EM_ phenotype cells with PSGL-1-deficiency, as indicated by expression of CD62L. Importantly, the accumulation of greater numbers of *Selplg^-/-^* T cells was a result of increased survival and not proliferation, as shown in our previous study of LCMV Clone 13 infection (9). In addition, *Selplg^-/-^* T cell survival was cell-intrinsic as both CD4^+^ and CD8^+^ virus-specific T cells exhibited greater persistence as memory cells in WT hosts after viral infection.

Consistent with this result, memory T cells from *Selplg^-/-^* mice had increased expression IL-7Rα and IL-2Rβ, components of the receptors that regulate responses to the γc cytokines IL-7, IL-2 and IL-15, which regulate T cell survival (27). These results corroborate a previous study showing that *Selplg^-/-^* T cells exhibit elevated homeostatic turnover in WT mice, and are in partial agreement with the finding in that study which showed that *Selplg* ^-/-^ T cells proliferate in response to high levels of IL-2 or IL-15 *in vitro*. Importantly, we did not find differences in expression levels of IL-7Rα on naïve T cells from WT and *Selplg* ^-/-^ mice (not shown), indicating that the inhibitory function of PSGL-1, which impacts the expression levels of γc cytokine receptors, occurs in the context of T cell activation.

The effects on survival that led to an overall increase in the availability of effector T cells after infection thereby increased the numbers of cytokine-producing T cells in *Selplg^-/-^* mice that were maintained during memory development. Polyfunctionality was also exhibited by greater frequencies of T cells from *Selplg* ^-/-^ mice compared to those from WT mice. To a large extent, this outcome was linked to a more rapid decrease in PD-1 expression on virus-specific *Selplg* ^-/-^ T cells. PD-1, which is induced T cells shortly after activation, was previously shown to inhibit the P14 effector T cell response during the first few days after LCMV Arm infection (5). Since virus-specific WT and *Selplg* ^-/-^ cells expressed comparable high levels of PD-1 at the peak of viral replication at 5dpi, but then more quickly lost expression, our results imply that PSGL-1-deficiency supports a more rapid effector to memory transition. The results also show that PSGL-1 does not directly regulate PD-1 at the peak of the viral replication, and possibly thereafter. Our finding that PD-1 expression is retained by virus-specific cells for an extended period after viral clearance suggests that maintenance of PD-1 is not likely to be due to residual antigen. However, PD-1 expression is linked to antigen recognition by the TCR, and different affinities might be expected lead to changes in PD-1 expression as shown by the different LCMV epitopes examined. It is possible that PSGL-1 signals sustain PD-1 expression on WT cells (NP396^+^ CD8^+^ cells as well as WT GP66^+^ CD4^+^ cells), but this will require further study. Overall, we conclude that PSGL-1 could potentially contribute to effector T cell death during the contraction phase of the response by sustaining greater activation, and thus PD-1 expression.

Other studies of PD-1 regulation of T cell anti-viral responses support the conclusion that inhibitory signals function to limit the magnitude of effector T cell responses. In response to acute infection with Friend’s Virus, PD-1- or PD-L1-deficiency resulted in enhanced effector CD8^+^ T cell development that was associated with greater survival and polyfunctionality (28). A study of acute vaccinia virus infection showed that PD-1-deficiency not only dramatically increased virus-specific T cell recovery at the effector and memory stages, but in contrast to our study, that memory effector CD8^+^ T cell responses after adoptive transfer and challenge of naïve hosts were also significantly greater. Yet another study of PD-1-axis deficiency with influenza virus infection showed greater effector responses, but more dramatic effector contraction and decreased memory CD8^+^ T cell function (8). Together the data imply that the fine tuning of T cell effector responses by inhibitory receptors is influenced by the tissue sites of infection, viral loads, and/or inflammatory conditions engendered by individual viruses.

Our results showing that effector generation is greater in *Selplg* ^-/-^ mice indicates that PSGL-1 dampens the response with LCMV Arm infection. Greater numbers of virus-specific effector CD4^+^ and CD8^+^ T cells in *Selplg* ^-/-^ mice were found early after infection, and MPEC phenotype T cells with significantly elevated expression of IL-7R predominated by 8dpi. Interestingly, PD-1 blockade during LCMV Arm infection also demonstrated increased numbers of MPECs possibly due to earlier viral control (5). Thus, we anticipated that the virus might be more rapidly controlled in *Selplg* ^-/-^ mice thereby limiting the extent of antigen stimulation and hastening the effector to memory transition. However, we did not detect differences in viral titers in the spleens of WT and PSGL-1-deficient mice at 4dpi, implying that differences in the regulation of T cell differentiation with PSGL-1-deficiency rather than earlier viral control had a greater impact on the memory development. Our finding of increased expression of CD62L on *Selplg* ^-/-^ T cells at this time further supports an interpretation that differentiation of T_CM_ phenotype cells with memory progenitor potential (29) are favored with PSGL-1-deficiency.

Although strong TCR stimulation is typically identified with terminal T cell differentiation (30), a recent study demonstrated that greater TCR stimulation can favor T_CM_ differentiation when coupled to high levels of Bim and Bcl2 (31), which would not be dependent on differences in viral loads. However, it is also possible that PSGL-1-deficiency allows for greater activation of SLECs due to more limited PD-1 expression, thereby driving their terminal differentiation and promoting increased representation of MPECs. In contrast to our results, a recent study identified a key role for PD-1 in combination with LAG-3 in maintaining memory CD8^+^ T cell precursors early during infection (7). As additional evidence for roles of inhibitory receptors in regulating T cell responses, both CTLA-4 and PD-1 were found to restrain aberrant effector T cell differentiation and profound inflammation that occurs with their genetic deficiency either early or late in life, respectively (32). However, CTLA-4 primarily prevented the generation of aberrant CD4^+^ T cell phenotypes whereas PD-1 restricted CD8^+^ T cell differentiation. It is noteworthy that PSGL-1 appears to regulate both CD4^+^ and CD8^+^ T cells comparably with respect to survival and function, and we do not find that *Selplg* ^-/-^ mice develop spontaneous inflammatory responses or signs of autoimmunity as do mice that are deficient in PD-1 and CTLA-4.

Implicit in our results is the conclusion that limiting PSGL-1-signaling during T cell priming after infection or vaccination would favor a generation of a larger memory cell pool, but we were unable to identify a PSGL-1 blocking reagent to directly test this concept. Our data showing significantly better functionality of effector and persisting memory *Selplg* ^-/-^ CD8^+^ T cells in response to TCR stimulation *in vitro* further support a conclusion that the capacity for high quality memory responses is potentially improved when the contributions of PSGL-1-dependent inhibition are removed. However, although *Selplg^-/-^* mice sustained greater numbers and frequencies of effector and memory T cells throughout the course of viral infection, with an adoptive transfer approach to prime WT and *Selplg* ^-/-^ T cells together in a PSGL-1-sufficient environment by LCMV Arm infection, they failed to mount a detectable memory effector response to a secondary viral challenge in naïve hosts as indicated by a failure to recover *Selplg* ^-/-^ T cells compared to WT T cells. This outcome reveals that despite an intrinsic survival advantage of *Selplg^-^* ^/-^ T cells even under conditions of competition with WT cells during their responses to the virus in WT hosts, the inhibitory effects of PSGL-1 resulted in a memory population with greater potential for secondary effector responses. Our results indicate that PSGL-1 can be a critical inhibitor of memory effector T cell responses as was also recently shown with the combined deficiency of both PD-1 and LAG-3 on memory CD8^+^ T cells with LCMV Arm where deficiency in these receptors supported greater recovery of effector T cells (7). We propose that *Selplg* ^-/-^ memory T cells may be less inhibited than their WT memory counterparts, as indicated by their increased cytokine responses to peptide restimulation *in vitro*. In the context of TCR signals, diminished inhibition could result in enhanced responses to stimulatory signals (e.g., CD28 or proinflammatory cytokines) that would potentially be detrimental to memory T cell survival and ultimately their function if overstimulation occurred. Importantly, we did not detect greater susceptibility of *Selplg* ^-/-^ memory T cells to TCR/peptide-mediated activation-induced cell death *in vivo*, suggesting that the virus challenge is coupled to their impaired memory effector responses in WT hosts.

Since we previously showed that PSGL-1 inhibitory signaling promoted the generation of exhausted T cells (9), it is significant that this inhibitory axis also regulates T cell differentiation during acute viral infection. Overall our results indicate that PSGL-1 is a fundamental negative regulator of T cells that ensures that TCR signals are fine-tuned to promote the generation of memory T cells that respond optimally to confer protection upon re-exposure to previously encountered viral pathogens. But importantly in the acute setting, PSGL-1 could be uniquely required to limit the responses of memory T cells, which are considered to be more readily activated by lower levels of antigen stimulation and costimulation than naïve T cells. Further studies will be required to identify how PSGL-1 contributes to the integration of co-inhibitory and co-stimulatory signals, cytokines, and the strength of TCR signals that together dictate the quantity and quality of the memory T cell pool.

## Data Availability Statement

The raw data supporting the conclusions of this article will be made available by the authors, without undue reservation.

## Ethics Statement

The animal study was reviewed and approved by IACUC committees at the Sanford Burnham Prebys Medical Discovery Institute and at the University of California, Irvine.

## Author Contributions

RT and LB initiated and designed the study as well as analyzed and interpreted experiments. EN and MH helped with *in vivo* experiments at UC Irvine, CS contributed to the *in vivo* experiments and performed virus analyses at SBP. RT and LB wrote the manuscript. RT and LB are corresponding authors. All authors contributed to the article and approved the submitted version.

## Funding

The authors would like to acknowledge funding from the NIH Grant R01 AI137239 to RT; NIH T32 AI007319 to EN, and R01 AI106895 and R21 AI15916 to LB. The work at SBP was also supported by the NIH NCI Cancer Center Support Grant P30 CA030199.

## Conflict of Interest

The authors declare that the research was conducted in the absence of any commercial or financial relationships that could be construed as a potential conflict of interest.
