# Supplementary material for: PSGL-1 Is a T Cell Intrinsic Inhibitor That Regulates Effector and Memory Differentiation and Responses During Viral Infection
Source: Front Immunol. 2021 Jul 13;12:677824. doi: 10.3389/fimmu.2021.677824 (PMC8314012; doi:10.3389/fimmu.2021.677824)
Supplement: Supplementary file 1 [file DataSheet_1.pdf]

Supplemental Figures for:

PSGL-1 is a T cell intrinsic inhibitor that regulates effector and memory differentiation and responses during viral infection

Roberto Tinoco[1]*, Emily N. Neubert[1], Christopher J. Stairiker[2], Monique L. Henriquez[1], and Linda M. Bradley[2]*

[1]Department of Molecular Biology and Biochemistry, University of California, Irvine, Irvine, CA 92093

[2]Infectious and Inflammatory Disease Center, NCI Designated Cancer Center, Sanford Burnham Prebys Medical Discovery Institute, La Jolla, CA 92037

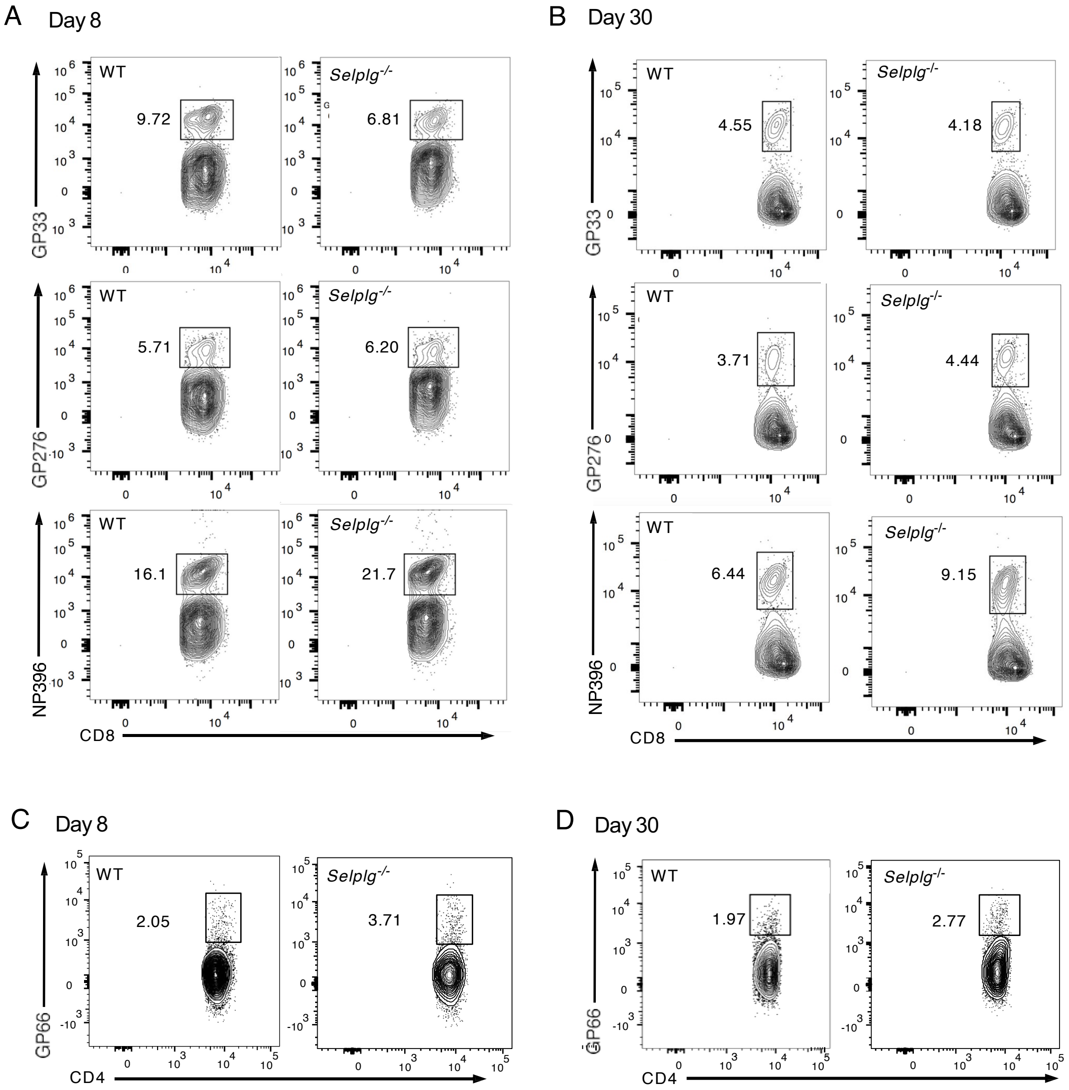

Supplemental Figure 1: Recovery of virus-specific T cells following LCMV infection.WT and *Selplg*$^{-/-}$ mice were infected with LCMV Armstrong and the frequencies of T cells enumerated at the indicated time points. For CD8^{+} cells, representative flow cytometry plots of CD8^{+} tetramer^{+} cells are in shown in (**A**) and (**B**). For CD4^{+} cells, representative flow cytometry of CD4^{+} tetramer^{+} cells are shown in (**C**) and (**D**). Data are representative of four independent experiments (n = 5 or more mice/group).

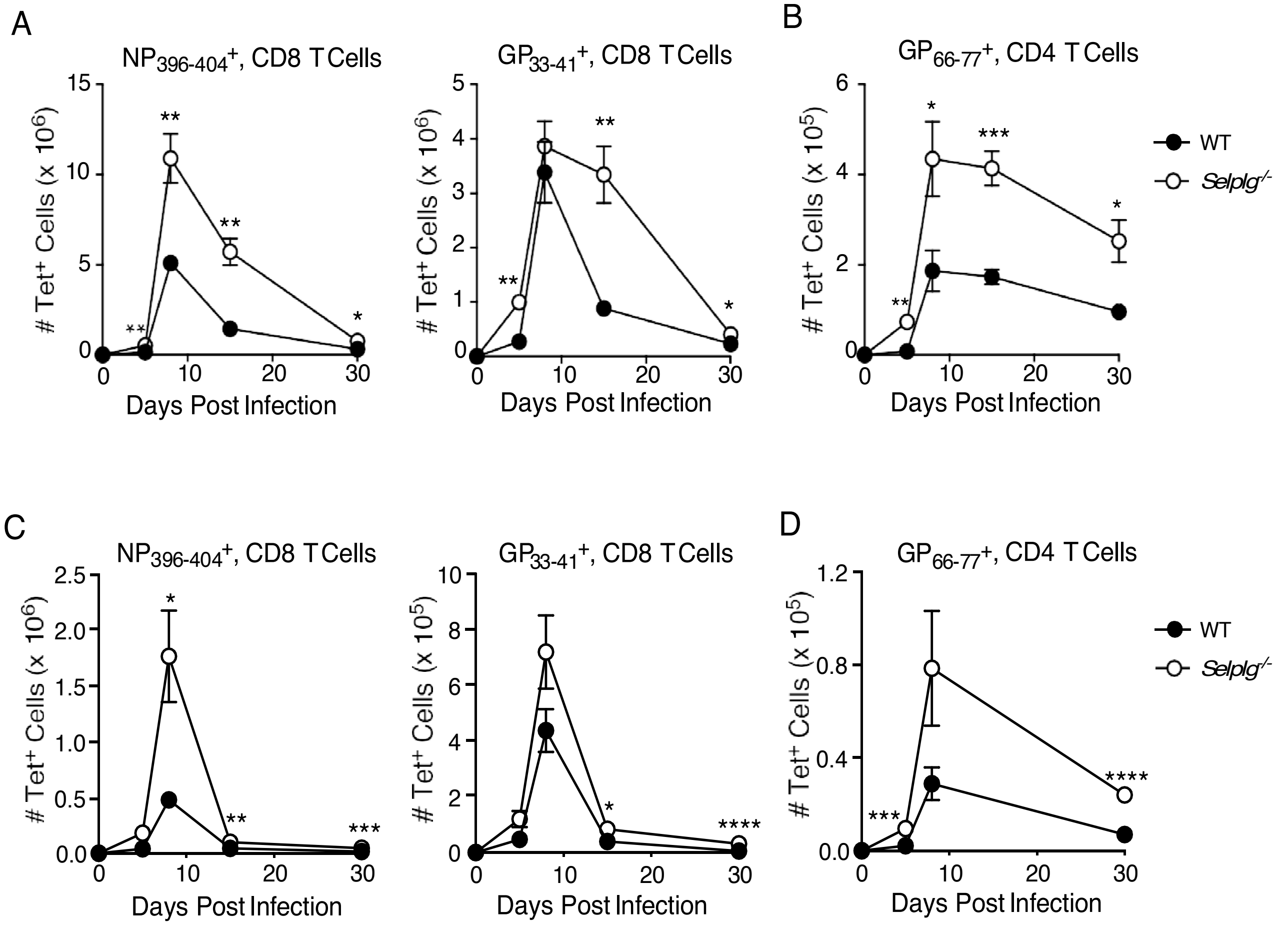

Supplemental Figure 2: Increased accumulation of *Selplg*$^{-/-}$ T cells during LCMV infection. WT and *Selplg*$^{-/-}$ mice were infected with LCMV Armstrong and T cells were enumerated at the indicated time points. CD8^{+} tetramer^{+} cells from the spleens are in shown in (**A**) and CD4^{+} tetramer^{+} cells from the spleens are shown in (**B**). (**C**) and (**D**) show tetramer^{+} CD8^{+} and CD4^{+} T cells from pooled peripheral lymph nodes from the same mice (n = 5/group). Graphs show the mean ± SEM. **P* < 0.05, ***P* < 0.005, ****P* < 0.001, *****P* < 0.0001 by unpaired two-tailed *t*-test at each time point

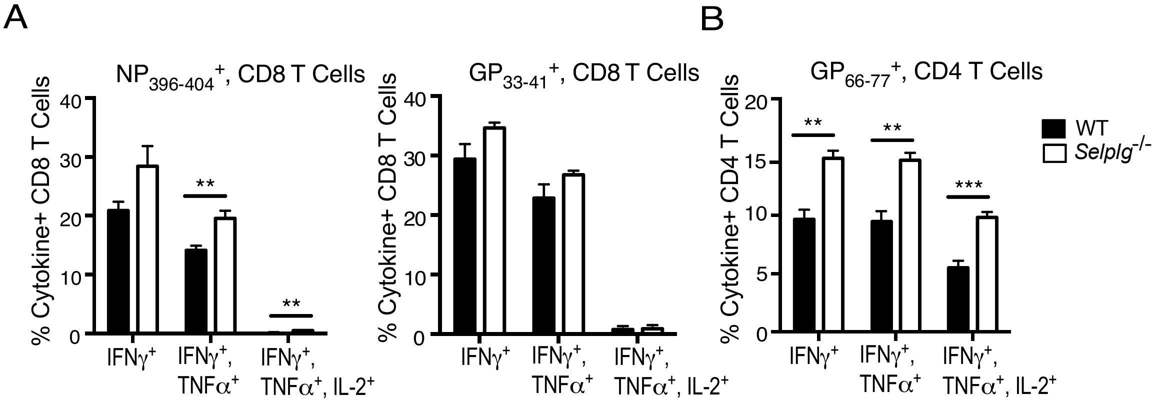

Supplemental Figure 3: Increased frequencies of cytokine producing virus-specific, *Selplg*$^{-/-}$ T cells. WT and *Selplg*$^{-/-}$ mice were infected with LCMV Armstrong and the frequencies of cytokine producing Tetramer^{+} CD8^{+} (**A**) and CD4^{+} (**B**) T cells were assessed by intracellular staining at 8 days after infection. n = 5/group. Graphs show the mean ± SEM. *$P < 0.05$, **$P < 0.005$, ***$P < 0.001$, by two-tailed *t*-test.

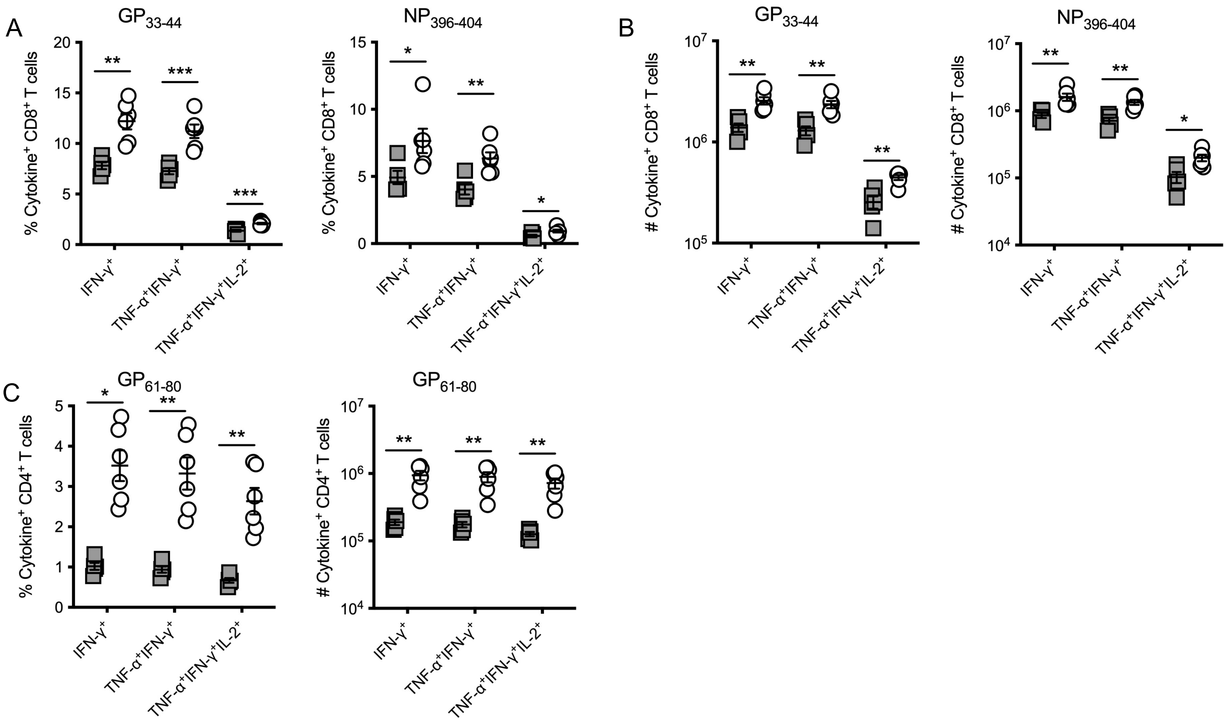

Supplemental Figure 4: Increased accumulation of functional virus-specific memory T cells in *Selplg*$^{-/-}$ mice. WT and *Selplg*$^{-/-}$ mice were infected with LCMV Armstrong and the spleens were isolated at 48dpi. Splenocytes were stimulated with the indicated viral peptides and the frequencies (**A**) and numbers (**B**) of cytokine^{+} CD8^{+} T cells and (**C**) CD4^{+} T cells are shown. Data are representative of one experiment (n = 5 or more mice/group). Graphs show the mean ± SEM. $*P < 0.05$, $**P < 0.005$, $***P < 0.001$, $****P < 0.0001$ by two-tailed *t*-test.

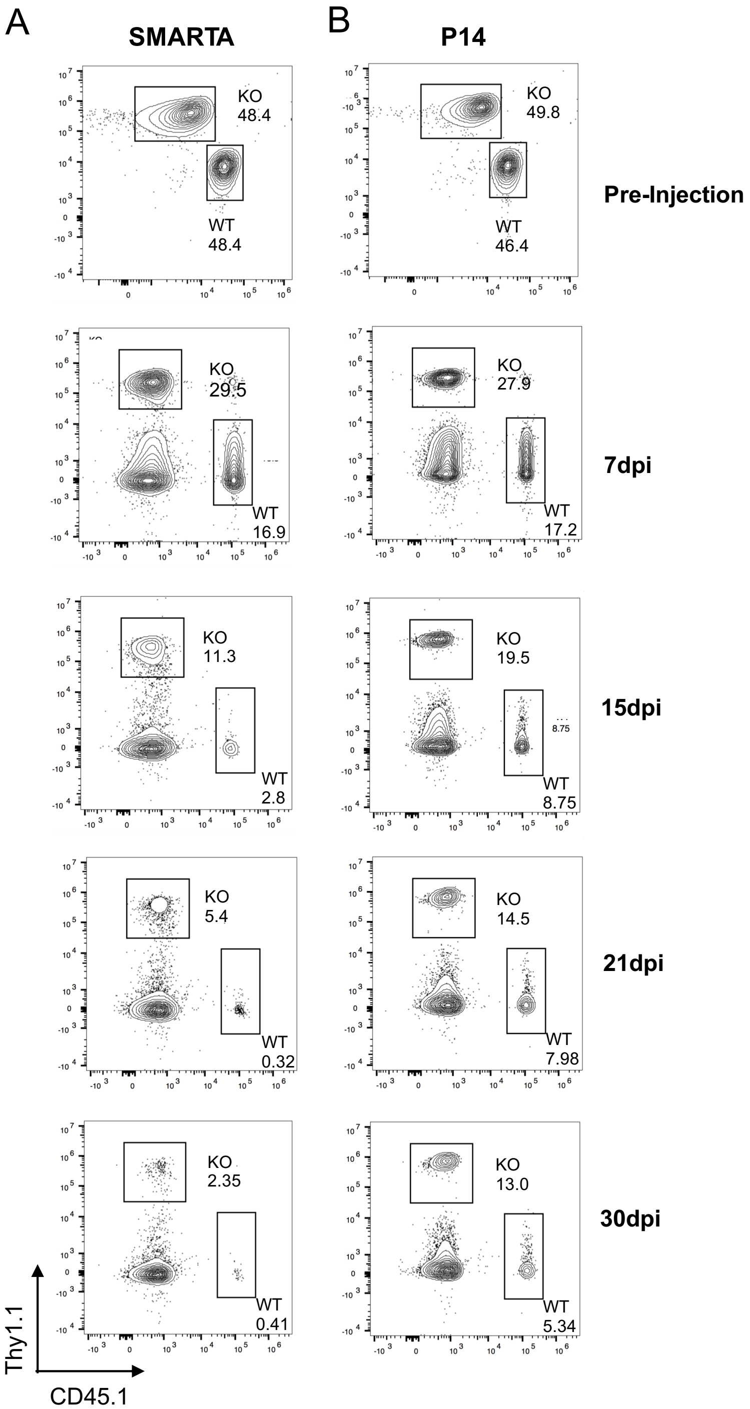

Supplemental Figure 5: Greater intrinsic decay of WT T cells compared to *Selplg*$^{-/-}$ T cells after virus infection. WT (CD45.1) and *Selplg*$^{-/-}$ (Thy1.1) SMARTA (**A**) or P14 (**B**) cells were isolated from spleens and were co-transferred in a 1:1 ratio by i.v. injection into naïve WT hosts that were then infected with LCMV Arm. Representative data showing the recovery of donor cells within the $CD4^{+}$ (SMARTA) or $CD8^{+}$ (P14) T cell populations at the indicated times after infection.

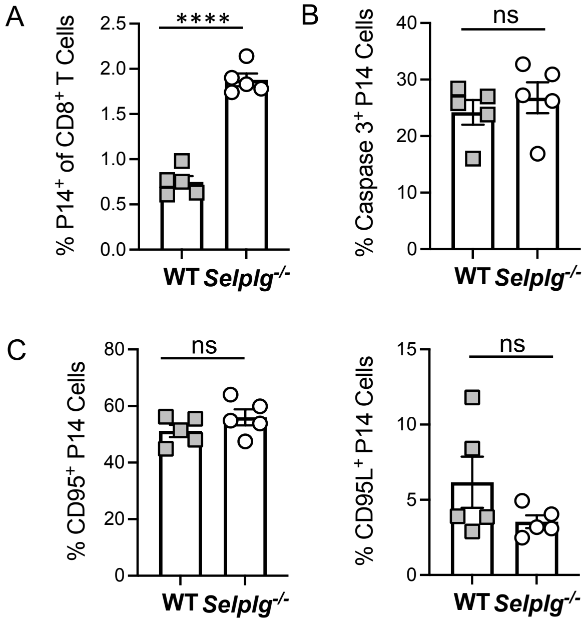

Supplemental Figure 6: Susceptibility to cell death in response to TCR stimulation does not differ between WT and *Selplg*$^{-/-}$ T cells. WT (CD45.1) and *Selplg*$^{-/-}$ (Thy1.1) P14 cells were isolated from spleens and were co-transferred by i.v. injection in a 1:1 ratio (5000 cells each) into naïve WT hosts (n = 5) that were then infected with LCMV Arm by i.p. injection. At 30 days post infection, the mice were injected i.v. with 50 µg of $GP_{33\text{-}41}$ peptide and 25 µg LPS. P14 cells were assessed for (**B**) intracellular active Caspase 3, and (**C**) for expression of CD95 and CD95L. The data are representative of 2 independent experiments. ****$P < 0.0001$ by two-tailed *t*-test.
